# An Intraoperative Episode of Severe Type II Renal Tubular Acidosis in an 83-Year-Old Woman

**DOI:** 10.7759/cureus.31786

**Published:** 2022-11-22

**Authors:** Emily Bergbower, Kelly Poe, Kiran Kaur, Robert Noorani

**Affiliations:** 1 Anesthesiology, University of Maryland Medical Center, Baltimore, USA

**Keywords:** perioperative management, intraoperative resuscitation, radical neck dissection, renal tubular acidosis type 2, anesthesiology

## Abstract

Type II renal tubular acidosis (RTA) is a rare defect in bicarbonate transport that can cause serious metabolic derangements. We report the case of a spontaneous, isolated intraoperative episode of severe type II RTA in an elderly woman who presented for radical neck dissection, mandible excision, and flap creation. Intraoperatively, she developed a stark metabolic acidosis with hypokalemia, progressively worsening base excess, and prolific urine output. Aggressive resuscitation with bicarbonate corrected all metabolic abnormalities. There was no identifiable trigger and the patient was successfully discharged with no further recurrences during hospitalization. The inability to identify the clinical presentation of RTA perioperatively can lead to poor outcomes.

## Introduction

The kidneys play a critical role in maintaining acid-base homeostasis through the regulation of bicarbonate resorption and excretion. Bicarbonate acts as a physiologic buffer that contributes to maintaining the plasma pH within a tight range to maximize physiological function [1]. Bicarbonate levels are primarily controlled by epithelial cells in the proximal renal tubule via unique transporters such as the electrogenic sodium bicarbonate cotransporter (NBCe1) [2].

Renal tubular acidosis (RTA) occurs when the kidneys fail to maintain the acid-base balance secondary to a defect in either H^+^ excretion or bicarbonate reabsorption. The three major types include type I distal RTA, type II proximal RTA, and type IV hyperkalemic RTA. Type I is characterized by impaired acid (H^+^) secretion, type II by the inability to reabsorb the filtered bicarbonate load, and type IV by abnormalities in H^+^ excretion and K^+^ balance [1,3]. Here, we present the case of an 83-year-old woman who experienced a spontaneous, isolated, and transient episode of type II proximal RTA intraoperatively that was treated to complete resolution in the operating room. Isolated episodes of type II RTA are very rare and, to the best of our knowledge, this is the first reported intraoperative occurrence.

## Case presentation

An 83-year-old ASA 3 woman with a history of atrial fibrillation, coronary artery disease (CAD), and lower lip squamous cell carcinoma (SCC) initially presented to the otolaryngologist with bulky bilateral lymphadenopathy, which was identified as SCC via biopsy. The tumor encased the right mandible and was noted to have broken through the skin on the right side of the neck. After assessment in the clinic, the patient was scheduled for an elective bilateral radical neck dissection, right mandible resection, tracheostomy with a Bjork flap, and left scapular chimeric osteocutaneous free flap.

On the day of surgery, the patient presented to the pre-operative waiting area from home. She was afebrile and mildly tachycardic with a heart rate (HR) of 103, BP of 94/62, and oxygen saturation (SpO2) of 97% on room air. Her metabolic equivalents (METs) were approximately 4-5. Morning laboratory results were significant for a WBC count of 15.6, sodium of 128, and chloride of 91. All other laboratory results were within normal limits, including an ionized calcium of 1.20 and a potassium (K^+^) of 3.9. The patient's tumor caused dysphagia and her preoperative sodium of 128 was presumed to be related to reduced oral intake ahead of surgery. It was not deemed clinically significant enough to delay resection. Upon arrival at the OR, the patient was pre-oxygenated with a face mask and general anesthesia was induced with 90 mg of propofol, 50 mg of 1% lidocaine, 2 mg of midazolam, and 25 mcg of fentanyl. She was paralyzed with 20 mg of rocuronium and 80 mg of succinylcholine, with rocuronium being given as a precurarizing dose to reduce some of the negative side effects of succinylcholine. Intubation was achieved in one attempt with a 6.0 endotracheal tube (ETT) and was uncomplicated, as the airway itself was anatomically unremarkable. She had two large-bore peripheral IVs placed for access as well as a radial arterial line for close hemodynamic monitoring and frequent sampling of blood.

In the first hour of surgery, the patient received several 100 mcg boluses of phenylephrine for blood pressure support as well as cefazolin and metronidazole for antibiotic prophylaxis. She was also given 4 mg of dexamethasone. An initial arterial blood gas (ABG) revealed a pH of 7.4, partial pressure of carbon dioxide (pCO_2_) of 34, partial pressure of oxygen (pO_2_) of 463, bicarbonate (HCO_3_) of 21, and a base excess of -2.8. She was noted to be hypokalemic at 2.7 and hypocalcemic at 1.03, a marked change from her normal preoperative labs of K^+^ 3.9 and calcium 1.20 collected on the morning of surgery. Subsequently, she was aggressively repleted with 40 mEq of K^+^ and 1 g of calcium.

After the third hour, another ABG was collected, which showed mild worsening of her metabolic acidosis with pH 7.4, PCO_2_ 31, HCO_3_ 19, and a base excess of -4.3. Potassium remained low at 3.0 but calcium was appropriately repleted. The patient had received 2L of crystalloid and put out 2L of urine. At the fourth hour of surgery, another ABG revealed a severe drop in bicarbonate to 14, and an increasingly negative base excess of -8.6. Potassium decreased again to 2.7, even after 50 mEq of repletion, and calcium dropped to 0.96 despite a total of 2.5g administered in divided doses. The patient received another 2L of crystalloid and urine output remained high at 500-600 mL per hour. At hour seven, the pH remained normal at 7.37 with hyperventilation to an end-tidal carbon dioxide (EtCO_2_) of 25, HCO_3_ of 14, and a base excess of -9.7. Despite 80 mEq of potassium, the patient remained mildly hypokalemic at 3.3 and hypocalcemic at 1.01. The patient received another 30 mEq of potassium, 1.5 g of calcium, and 2L of crystalloid with increasing urine output to approximately 900 mL per hour. At hour eight, the pCO_2_ was 20, base excess worsened to -12.0, and bicarbonate dropped to 11. Hypokalemia and hypocalcemia remained notable.

Differential diagnosis

Initially, our differential diagnosis focused very broadly on the causes of severe hypokalemia. Gastrointestinal (GI) loss of potassium was ruled out as the patient did not have pre-operative vomiting and diarrhea, a history of malabsorption, or a nasogastric tube set to suction. Poor dietary intake was also ruled out as an underlying cause given her normal pre-operative lab values and history. An intracellular shift of potassium from metabolic alkalosis, insulin excess, or intoxication (theophylline, barium, caffeine) was also ruled out based on ABG results, history, and medication administration. Finally, the patient was not on dialysis.

Our differential then shifted to focus on renal losses as the underlying cause of her laboratory derangements. The patient notably had no history of mineralocorticoid excess, diuretic use, or recent treatments with aminoglycoside antibiotics. Osmotic diuresis was an unlikely source of potassium loss as the patient had no history of diabetes mellitus and maintained normal glucose values throughout the perioperative period. At this point, we began to consider the possibility that the patient was experiencing new-onset RTA, either type I or type II. Case reports do exist of spontaneous onset cases with no clear cause, especially among pregnant women in the third trimester. However, no spontaneous episode has ever been reported intraoperatively to our knowledge.

Outcome and follow-up

The decision was made to rapidly bolus the patient with 150 mEq of sodium bicarbonate. Approximately 45 minutes after this bolus, during hour nine, an ABG revealed a corrected bicarbonate of 26 and a base excess of 2.1. The patient's potassium normalized to 4.7 with a calcium of 1.31. The patient no longer required respiratory compensation through hyperventilation, and the pCO_2_ returned to baseline. Urine output slowed to 200 mL per hour. All other labs collected intraoperatively after this point were unremarkable and the surgery was completed without further issues. At the close of the case, the patient was taken to the surgical intensive care unit (SICU) and intubated for further management. She has no postoperative kidney issues and no further electrolyte derangements. ABG results over time are summarized in Table 1. Total fluids and electrolytes administered are summarized in Table 2. 

**Table 1:** Six summarized intraoperative ABGs with the associated hourly urine output The first five were collected prior to bolusing bicarbonate. The sixth ABG at hour 9 was run 45 minutes after the bicarbonate bolus. ABG: arterial blood gas; pCO_2_: partial pressure of carbon dioxide; pO_2_: partial pressure of oxygen; HCO_3_: bicarbonate

	Hour 1	Hour 3	Hour 4	Hour 7	Hour 8	Hour 9
pH	7.40	7.40	7.42	7.37	7.38	7.43
pCO_2_ (mmHg)	34	31	23	25	20	40
pO_2_ (mmHg)	463	212	218	209	206	186
HCO_3_ (mmol/L)	21	19	14	14	11	26
Base Excess (mmol/L)	-2.8	-4.8	-8.6	-9.7	-12.0	2.1
Potassium (mmol/L)	2.7	3.0	2.7	3.3	2.7	4.7
Calcium (mmol/L)	1.03	1.17	0.96	1.01	0.90	1.31
Urine output/hour (mL/hr)	110	350	550	700	925	200

**Table 2:** Total fluids and electrolytes administered throughout the entirety of the case pRBCs: packed red blood cells

	Case Total
Potassium	115 mEq
Magnesium	4g
Calcium	4.75g
Bicarbonate	150 mEq
Plasmalyte	9L
Albumin	500mL
pRBCs	4U
Total Urine Output	5.4 L

## Discussion

The kidneys play a significant role in the maintenance of acid-base homeostasis through tight regulation of acid secretion and bicarbonate reabsorption. RTA will develop when some loss of tubular function prevents the kidney from maintaining this tight regulation [4,5]. Initial laboratory analysis will reveal a normal anion gap and a hyperchloremic metabolic acidosis often in the setting of preserved baseline kidney function [1,6-7]. RTA is divided into three subtypes, which are each characterized by the inability of the renal tubule to reabsorb bicarbonate or to secrete hydrogen ions [5].

**Figure 1:**
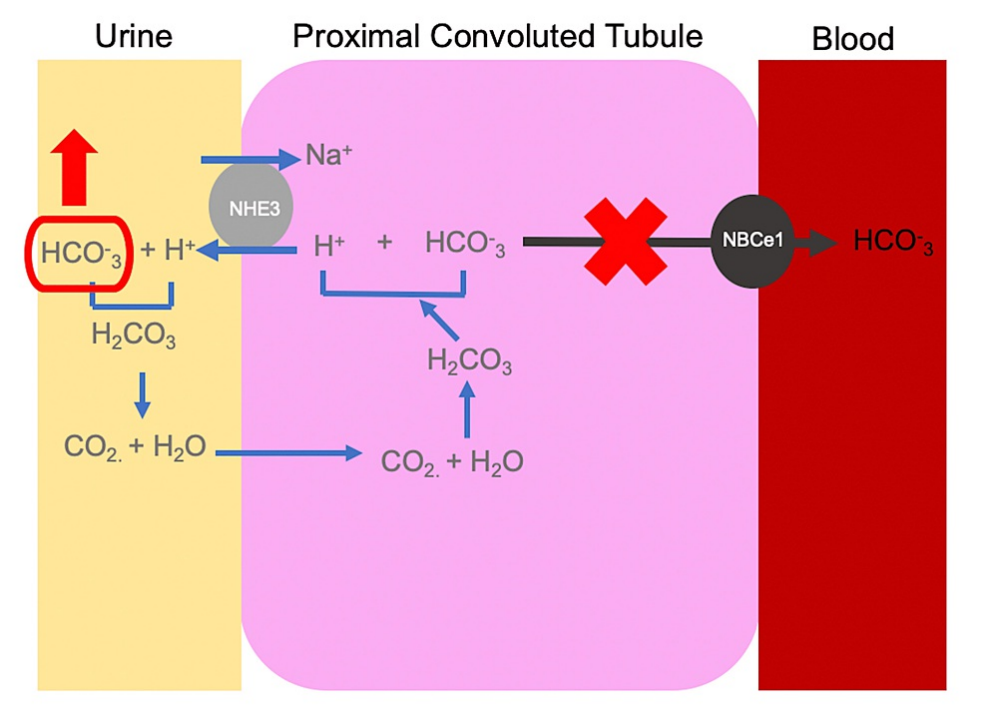
The proximal tubule defect leading to type II RTA Here, the baseline defect lies in the ability of the proximal tubule to reabsorb bicarbonate (red “X”). As such, increasing amounts of bicarbonate are excreted in the urine, leading to metabolic acidosis. Notably, urine can be acidified via H+ transport in intercalated cells, but this is often not enough to compensate. RTA: renal tubular acidosis

Here, we present a likely case of spontaneous, isolated type II proximal RTA intraoperatively. Type II RTA is characterized by the inability of the proximal tubule to resorb bicarbonate, which is responsible for approximately 80% of the daily filtered load [3]. At baseline, epithelial cells of the proximal tubule generate new bicarbonate to replace what’s lost in the urine and also reabsorb bicarbonate from the glomerular filtrate via the NBCe1 cotransporter (Figure 1) [2,8-10]. Defective bicarbonate handling is what leads to low plasma HCO_3_, low urine pH, hypokalemia, and increased bicarbonate wasting in urine [1,2].

Type II proximal RTA can be inherited, acquired, or idiopathic [5]. In our case, the patient had not displayed any physical or biochemical traits throughout her life, consistent with the inherited forms of proximal RTA. This presentation was also unlikely to be acquired or drug-induced, as is common when proximal RTA presents with Fanconi’s Syndrome. She was not taking carbonic anhydrase inhibitors nor had she been taking nucleoside reverse transcriptase inhibitors (NRTIs), cisplatin/oxaliplatin, valproic acid, or aminoglycosides [1,3]. There was no known heavy metal exposure. Her preoperative labs were normal, and she reported no symptoms consistent with type II RTA, such as muscle weakness or paralysis, in the preoperative interview. Thus, it seems more likely than not that the case presented is idiopathic.

When idiopathic in origin, type II RTA is typically a sporadic event that resolves with treatment. These episodes are very rare but have been reported in the literature, especially among pregnant women. In these cases, type II RTA was diagnosed in the third trimester and completely resolved after delivery [11,12]. The etiology remains unclear. The present case appears to be the first time a transient intraoperative episode has been reported in the literature.

This report has a notable limitation in that we were not able to diagnostically confirm beyond a reasonable doubt that this is type II RTA. A definitive diagnosis of type II RTA requires a NaHCO_3 _loading test, which could not be obtained given our window of time with the patient, lack of access to the main laboratory, and quick resolution. We were also unable to confirm a urine pH, which would have further bolstered the diagnosis [3]. However, overall, this presentation seems more consistent with type II RTA than type I RTA. We note that this patient exhibited a severe hypokalemic metabolic acidosis with intravascular volume depletion and increasing quantities of urine output, all consistent with type II. Additionally, this patient required an aggressive quantity of alkali therapy to reverse the scenario, which is also more consistent with type II.

Here, we have presented an unusual episode of spontaneous, isolated, and possibly idiopathic type II RTA. This patient was successfully treated with bicarbonate to complete resolution in the OR. The underlying causative factor is unclear, similar to cases observed among pregnant women. We posit that this case provides a useful learning opportunity, regarding a rare presentation, for physicians handling care during the perioperative period.

## Conclusions

This case highlights four pertinent learning points. First, severe hypokalemia can be gastrointestinal or renal in origin and possibly caused by intracellular shifts. Intraoperatively, patients should be aggressively repleted as the differential is narrowed. Second, spontaneous episodes of type II RTA are rare and clinical manifestations often include hypokalemia, low bicarbonate levels, a non-anion gap metabolic acidosis, and high urine output. Hypocalcemia has been noted in several case reports. Third, definitive testing to differentiate type I from type II requires a urine pH and a NaHCO_3_ loading test. Finally, with regards to treatment, first-line therapies include bicarbonate replacement through bolusing or an infusion if necessary, as well as electrolyte repletion.
